# Weaning Induced Hepatic Oxidative Stress, Apoptosis, and Aminotransferases through MAPK Signaling Pathways in Piglets

**DOI:** 10.1155/2016/4768541

**Published:** 2016-10-11

**Authors:** Zhen Luo, Wei Zhu, Qi Guo, Wenli Luo, Jing Zhang, Weina Xu, Jianxiong Xu

**Affiliations:** School of Agriculture and Biology, Shanghai Jiao Tong University, Shanghai Key Laboratory of Veterinary Biotechnology, Shanghai 200240, China

## Abstract

This study investigated the effects of weaning on the hepatic redox status, apoptosis, function, and the mitogen-activated protein kinase (MAPK) signaling pathways during the first week after weaning in piglets. A total of 12 litters of piglets were weaned at d 21 and divided into the weaning group (WG) and the control group (CG). Six piglets from each group were slaughtered at d 0 (d 20, referred to weaning), d 1, d 4, and d 7 after weaning. Results showed that weaning significantly increased the concentrations of hepatic free radicals H_2_O_2_ and NO, malondialdehyde (MDA), and 8-hydroxy-2′-deoxyguanosine (8-OHdG), while significantly decreasing the inhibitory hydroxyl ability (IHA) and glutathione peroxidase (GSH-Px), and altered the level of superoxide dismutase (SOD). The apoptosis results showed that weaning increased the concentrations of caspase-3, caspase-8, caspase-9 and the ratio of Bax/Bcl-2. In addition, aspartate aminotransferase transaminase (AST) and alanine aminotransferase (ALT) in liver homogenates increased after weaning. The phosphorylated JNK and ERK1/2 increased, while the activated p38 initially decreased and then increased. Our results suggested that weaning increased the hepatic oxidative stress and aminotransferases and initiated apoptosis, which may be related to the activated MAPK pathways in postweaning piglets.

## 1. Introduction

Weaning is abruptly stressful in the neonates' life, and that stress can result in growth retardation and susceptibility to diseases in mammals. These issues are especially serious in commercial swine husbandry during the first week after weaning, which often results in “postweaning stress syndrome (PWSD)” [1]. Previous studies have reported that weaning decreased intestinal digestive enzyme activities, damaged tight junction proteins, impaired immune response and barrier function, increased cytokines, and activated signaling pathways, both transiently and long term [2–5]. Our recent study indicated that weaning disrupted the physiologic equilibrium of oxidant and antioxidant and led to oxidative stress, eventually inducing enterocyte apoptosis and cell cycle arrest in the small intestine of postweaning piglets [6, 7].

The liver, which is located between the absorptive surfaces of the gastrointestinal tract, plays an important role in nutrients' metabolism and transformation. The liver's blood supply primarily originates from the intestine through the portal vein; so when the intestinal function is disrupted, an increase in intestinal permeability may contribute to the translocation of metabolites to the liver and impairment of liver function [8, 9]. Furthermore, the liver is more vulnerable to damage under particular kinds of stress [10]. The liver is a thermogenic organ that contains a large number of mitochondria in mammals, which is the place with high oxygen consumption and reactive oxygen species (ROS) formation [11, 12]. However, changes in hepatic function throughout the weaning period receive less attention from researchers compared to the intestine. One study showed that weaning induced endoplasmic reticulum stress in the liver of piglets [13]. Little data is available regarding weaning-induced oxidative injury, apoptosis, and loss of function in the potential mechanisms within the piglets' liver. The mitogen-activated protein kinases (MAPKs), the family of the serine-threonine protein kinases that transduce signals from the cell membrane to the nucleus, include extracellular signal-regulated kinases (ERKs), c-Jun N-terminal kinases (JNKs) and p38 and, in particular, participate in oxidative stress-induced cell apoptosis and proliferation [14]. MAPKs have been shown to be activated in an ischemic-injured ileum and in a weaning jejunum in pigs [5, 15]. Whether weaning results in MAPKs activation in the liver remains unknown.

The present objective was to study the immediate effects of weaning on the markers of hepatic oxidative stress, apoptosis, function, and MAPK signaling pathways in postweaning piglets and then to evaluate whether there was a novel, promising method for preventing weaning stress in human beings and domestic animals.

## 2. Materials and Methods

### 2.1. Animals, Diets, and Sampling

The experiment was conducted according to the guidelines of Shanghai Jiao Tong University Institutional Animal Care and Use Committee. A total of 120 7-day-old piglets (Duroc × Landrace) from 12 litters were randomly assigned by litter to two groups: the normal suckling (control group, CG) and the weaning group (WG), resulting in six litters per group. All piglets were kept with their sows in gestation crates in the same farrowing pens, while they suckled, and they had free access to the basal diet from d 7 to d 28. The dietary ingredients and nutrition levels are shown in Table 1. At d 21, 6 litters of piglets were randomly selected, weaned, and then moved to nursery pens; the others remained with their mothers without mixing any litters. Water was consumed* ad libitum*, and the temperature in nursery pens was about 30°C with a relative humidity of 50–70%. Pens were regularly cleaned of the manure, and the pig house was well-ventilated. At d 20 after birth (d 0), before the separation of the piglets into the two study groups, six piglets with a similar body weight from six different litters were randomly selected and sacrificed for testing (half males and half females). At d 1, d 4, and d 7 after weaning, one piglet of similar body weight from each litter was selected and sacrificed for testing. All the selected pigs were anaesthetized by intramuscular injection of sodium pentobarbital (Merck, Germany) (40 mg/kg BW). Liver tissues were sampled and immediately stored at −80°C for analysis of redox status, enzyme activities, and protein expression.

### 2.2. Determination of H_2_O_2_ and NO in Liver Tissues

The liver tissues were weighed and homogenized in an H_2_O_2_ lysis buffer (1 : 20, w/v) according to the manufacturer's instructions (Beyotime Biotech, Shanghai, China). The supernatants were gathered by centrifuging at 12,000 ×g for 10 min. Briefly, the sample solution (50 *μ*L) was incubated with reaction solution (100 *μ*L) at room temperature for 30 min, and then the absorbance was read at 560 nm. The H_2_O_2_ concentration was calculated by the standard curve made from the standard solutions.

NO production in tissues was measured by the Griess method according to the specification of the NO assay kit (Beyotime Biotech, Shanghai, China). Briefly, liver tissues were weighed and homogenized in a RIPA lysis buffer (1 : 10, w/v). The supernatants were gathered by centrifuging at 12,000 ×g for 10 min. The sample solution (40 *μ*L) and an equal volume of Griess reagents I and II were added to a 96-well microplate. The absorbance was measured at a wavelength of 540 nm. The NO concentration was calculated using a curve calibrated from sodium nitrite standards.

### 2.3. Determination of Lipid Peroxidation and Antioxidant Enzyme Activity

The activities of malondialdehyde (MDA) (Nanjing KeyGEN BioTech, Nanjing, China), the inhibitory hydroxyl ability (IHA), superoxide dismutase (SOD), glutathione peroxidase (GSH-Px) (Nanjing Jiancheng Bioengineering Institute, Nanjing, China) in the liver were determined through enzymatic colorimetric methods according to the commercial kits, respectively. Briefly, the concentration of MDA, which is an indicator of lipid peroxidation, was analyzed using the thiobarbituric acid (TBA) method to generate a colored product with an absorbance at 532 nm. The concentration of IHA was detected by the Fenton reaction method and the absorbance was read at 550 nm. Activity of SOD was determined using the hydroxylamine method, and absorbance was recorded at 550 nm. GSH-Px activity was expressed by measuring the reduction of glutathione per min after the subtraction of the nonenzymatic reaction. All absorbance levels were determined using a UV-visible spectrophotometer (Tongfang, Inc., China) as described by Zhu et al. [6].

### 2.4. ELISA for DNA Injury, Caspases, and Indices of Hepatic Function

The activities of 8-hydroxy-2′-deoxyguanosine (8-OHdG), caspase-3, caspase-8, caspase-9 (Nanjing Jiancheng Bioengineering Institute, Nanjing, China), aspartate aminotransferase transaminase (AST) and alanine aminotransferase (ALT), alkaline phosphatase (ALP), and gamma glutamyltransferase (GGT) (Shanghai Yuanye Bioengineering Institute, Shanghai, China) were determined using commercially available enzyme-linked immune sorbent assay (ELISA) kits. Briefly, liver tissues were homogenized in 0.9% of saline solution and then centrifuged at 12,000 ×g for 15 min, to release the enzymes into the solution. Then, the microplates were coated of with anti-8-OHdG, caspase-3, caspase-8, caspase-9, AST, ALT, GGT, and ALP, followed by detection with a horseradish peroxidase-labeled substrate after incubation for 10 minutes at 37°C. Absorbance values were then read in a spectrophotometer at 450 nm.

### 2.5. Western Blot Analysis

Liver samples were homogenized in 500 *μ*L of ice-cold RIPA lysis buffer (KGP703-100, KeyGEN Biotech, Nanjing, China), containing 1 mM of phenylmethylsulfonyl fluoride (PMSF, Amresco, Shanghai, China) and protease inhibitor cocktail tablets (05892791001, Roche, Germany) and incubating the suspension on ice for 30 min. The lysates were centrifuged for 10 min at 12,000 ×g, and the supernatants were collected. The protein content was measured using the BCA protein assay kit, according to the manufacturer's instructions (P0010, Beyotime Biotech, Shanghai, China). Tissue extracts (amounts equalized by protein concentration) were mixed with 5 × SDS-PAGE loading buffer (BL502A, Biosharp, US) and boiled for 3 min at 100°C. Protein (40 *μ*g) was electrophoresed in 10% SDS-PAGE gels and transferred to polyvinylidene difluoride (PVDF) membranes (0.45 *μ*m pore size, IPVH00010, Millipore, MA). The membranes were blocked for 2 h with 5% (w/v) skimmed milk powder (D8340, Solarbio, Shanghai, China) in Tris-Tween buffered saline (T-TBS) buffer [0.5 M NaCl (S7653, Sigma-Aldrich, Shanghai, China), 20 mM Tris (Amresco, Shanghai, China), pH 7.5, and 0.1% (v/v) Tween-20 (P7949, Sigma-Aldrich, Shanghai, China)], then washed three times with T-TBS, and incubated overnight at 4°C with primary antibodies following dilutions in 5% skimmed milk powder or BSA (0218054950, MP, US). The primary antibodies were JNK (1 : 2000, sc-571, Santa Cruz), p38*α* (1 : 200, sc-535, Santa Cruz), p-p38 (1 : 200, sc-7973, Santa Cruz), Bax (1 : 100, sc-493, Santa Cruz), Bcl-2 (1 : 200, sc-492, Santa Cruz), p-JNK (1 : 500, orb10951, Biorbyt Ltd, UK), ERK1/2 (1 : 1000, number 9102, Cell Signaling Technology), phospho-ERK1/2 (1 : 2000, number 4370, Cell Signaling Technology) (Thr202/Tyr204, Rabbit mAb) incubated overnight at 4°C, and then incubated with goat anti-rabbit (1 : 2000, ab97051, Abcam, UK) or goat anti-mouse IgG-HRP (1 : 2000, sc-2005, Santa Cruz) antibodies for 2 h. Image acquisition was performed on an enhanced chemiluminescence detection system (Tanon, Shanghai, China). Image J software was used to quantify the density of the specific protein bands.

### 2.6. Statistical Analysis

Data among groups both the CG and WG were tested for normal distribution using the statistical software SPSS 17.0 (SPSS Inc., Chicago, IL, US). If the data were not distributed normally, log transformation of variables was performed among treatment groups. No significant variance of data was found before the independent sample *t*-test method was conducted. All the data were presented as mean ± SD. *P* values < 0.05 were considered statistically significant.

## 3. Results

### 3.1. Concentrations of Free Radicals in the Liver

The concentrations of free radicals in liver homogenates are shown in Table 2. Compared with the CG, the content of H_2_O_2_ was significantly increased by 23.37% and 17.71% (*P* < 0.05) at d 1 and d 4 in the livers of the WG, but no difference was observed at d 7 between the CG and WG. Likewise, weaning increased the NO production at d 1 (by 25.23%) and d 7 (by 14.42%), while there was no significant difference at d 4.

### 3.2. Oxidant Injury and Antioxidant Enzyme Activity

**Figure 1:**
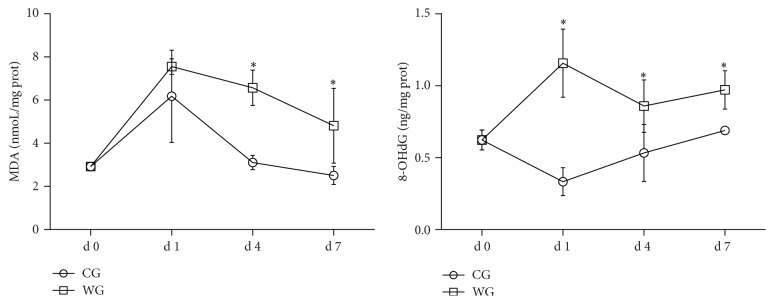
Oxidative injury obtained at d 0, d 1, d 4, and d 7 in the liver of WG compared with CG (*n* = 5). CG: control group; WG: weaning group. MDA: malondialdehyde (nmol/mg prot); 8-OHdG: 8-hydroxy-2-deoxyguanosine (ng/mg prot). ^*∗*^Means are significantly different (*P* < 0.05).

**Figure 2:**
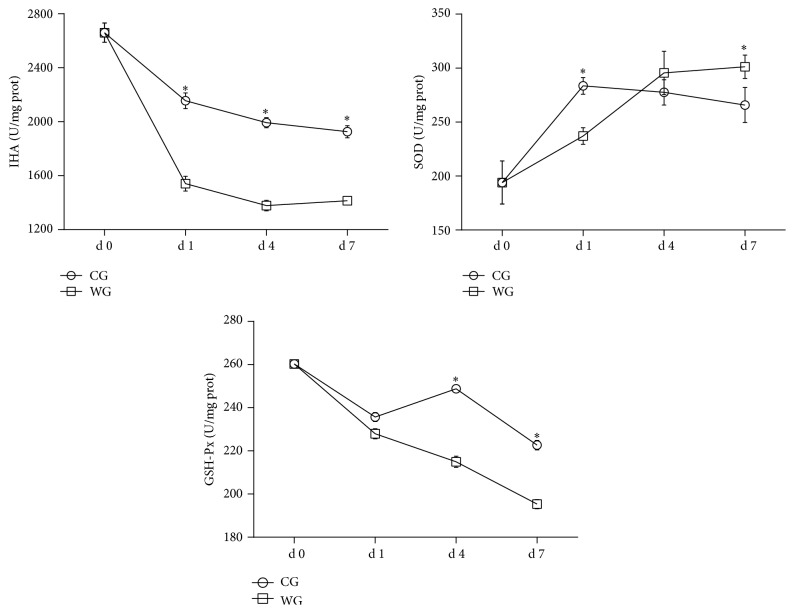
Antioxidant enzymes activities obtained at d 0, d 1, d 4, and d 7 in the liver of WG compared with CG. CG: control group; WG: weaning group. IHA: inhibitory hydroxyl ability (U/mg prot); SOD: superoxide dismutase (U/mg prot); GSH-Px: glutathione peroxidase (U/mg prot). ^*∗*^Means are significantly different (*P* < 0.05).

We measured the concentrations of MDA and 8-OHdG and monitored the activities of IHA, SOD, and GSH-Px in the liver homogenates of CG and WG (shown in Figures 1 and 2). As the marker of lipid peroxidation, hepatic MDA levels in WG were no different from that found in the CG at d 1, but hepatic MDA levels were significantly higher (*P* < 0.05) at d 4 and d 7. Compared with the CG, 8-OHdG, as an indicator of DNA injury, was significantly higher (*P* < 0.05) in the WG at d 1, d 4, and d 7. The concentration of inhibitory hydroxyl radical (IHA) was significantly higher (*P* < 0.05) in the WG at d 1, d 4, and d 7, although IHA showed a downward curve over that time period. Contrarily, the concentration of SOD in the WG was much less at d 1 and then significantly (*P* < 0.05) higher than that found in the CG at d 7. No significant difference was observed in GSH-Px activity at d 1 after weaning, but the value was significantly lowered (*P* < 0.05) at d 4 and d 7 in the WG when compared with the CG.

### 3.3. ELISA Results for Caspase Activity

**Figure 3:**
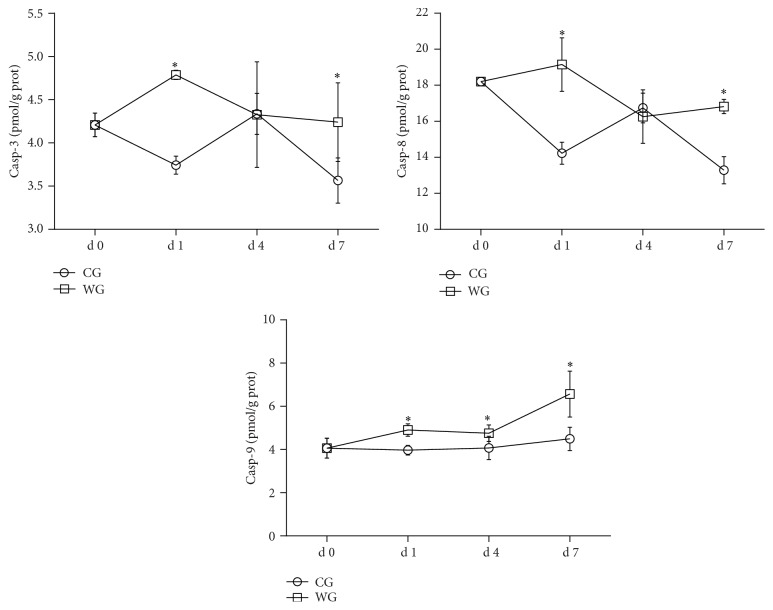
Caspase-3, caspase-8, and caspase-9 activities (pmol/g prot) at d 0, d 1, d 4, and d 7 in the liver of WG compared with CG. CG: control group; WG: weaning group. ^*∗*^Means are significantly different (*P* < 0.05).

As shown in Figure 3, the levels of caspase-3 and caspase-8 were significantly higher (*P* < 0.05) in the livers of WG than those in the CG at d 1 and d 7. No difference was observed in the concentrations of caspase-3 and caspase-8 at d 4. Compared with the CG, the WG experienced a significantly increased concentration of caspase-9 (*P* < 0.05) in the liver at d 1, d 4, and d 7.

### 3.4. ELISA Results for Enzyme Activities of Hepatic Function

**Figure 4:**
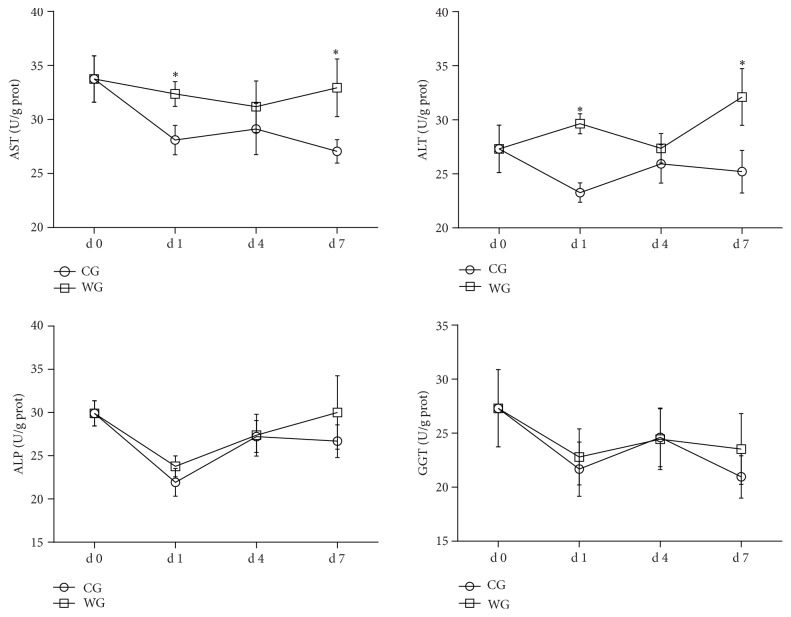
Indices of hepatic enzyme activities at d 0, d 1, d 4, and d 7 in WG compared with CG. CG: control group; WG: weaning group. AST: aspartate aminotransferase transaminase (U/g prot); ALT: alanine aminotransferase (U/g prot); ALP: alkaline phosphatase (U/g prot); GGT: gamma glutamyltransferase (U/g prot). ^*∗*^Means are significantly different (*P* < 0.05).

The effects of weaning on hepatic function at different time points are shown in Figure 4. The concentration of AST was significantly higher (*P* < 0.05) in WG than in the CG at d 1 and d 7 (by 5.35% and 8.08%, resp.). Meanwhile, the ALT activity was significantly increased (*P* < 0.05) in weaned piglets at d 1 (by 11.74%) and d 7 (by 10.29%). However, no significant differences were observed in the concentration of ALP and GGT in the WG when compared with the CG.

### 3.5. Protein Expression of MAPK Signaling Pathways and Regulator of Apoptosis

**Figure 5:**
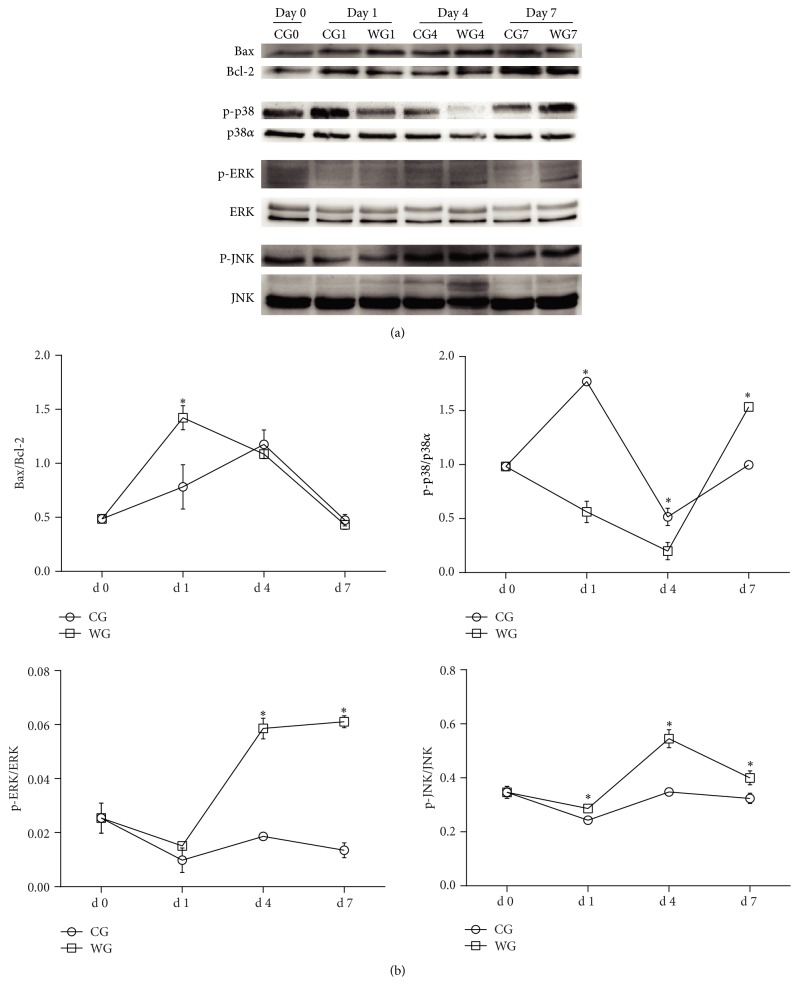
The protein expression of regulator of apoptosis and MAPK signaling pathways at d 0, 1, 4, and 7 in liver of WG compared with CG (a). The values are calculated as the ratios of Bax and Bcl-2 and their phosphorylation levels (p-JNK, p-p38, and p-ERK1/2) and the total levels of MAPK (b). CG: control group; WG: weaning group. ^*∗*^Means are significantly different (*P* < 0.05) (*n* = 3). Note: p38 [p38*α* (C20), SC-535, Santa Cruz] is recommended for detection of p38*α* as the total protein, while p-p38 [p-p38 (D-8) SC7379, Santa Cruz] is recommended for detection of p38*α*, p38*β*, and p38*γ* correspondingly.

We measured the effects of weaning on the protein expressions of regulators of apoptosis (ratio of Bax/Bcl-2) and MAPK signaling pathways (Figure 5). Results showed that the ratio of Bax/Bcl-2 was significantly higher (*P* < 0.05) in the WG at d 1, but there were no differences between the WG and the CG at d 4 or d 7. Compared with the CG, the ratio of p-ERK/ERK was increased (*P* < 0.05) at d 4 and d 7, while the ratio of p-JNK/JNK was increased (*P* < 0.05) at d 1, d 4, and d 7 in the WG. Weaning lowered (*P* < 0.05) the ratio of p-p38/p38*α* at d 1 and 4, but the value was significantly higher at d 7.

## 4. Discussion

Weaning is the result of multiple factors involving physiological, environmental, and psychological changes in piglets, which often induces oxidative stress in vivo [6, 16]. Oxidative stress is a systematic response involving the pathophysiology of many different disorders, including the hepatic redox status and function in piglets as shown in the present study [17].

H_2_O_2_ and NO are small, highly reactive molecules that play a dual role in normal cell progression, as both toxins and signaling molecules [18, 19]. In the present study, we found that NO and H_2_O_2_ were significantly increased in the liver homogenates of WG. The results were similar to our previous reports, which indicated that the concentrations of H_2_O_2_ and NO were increased in the serum, ileum, and colon of weaning piglets [6, 20]. Furthermore, to investigate whether the increased ROS led to oxidative injury caused by weaning, we determined the concentrations of MDA and 8-OHdG in the liver for both the CG and WG. MDA is derived by polyunsaturated fatty acid peroxidation and its contents have been considered a biological marker of lipid oxidative injury [21]. In this study, the concentration of MDA was significantly increased at d 4 and d 7 after weaning. However, our previous report found that the serum MDA increased only at d 14 after weaning, which indicated a postponed response [6]. We speculated that the liver was the major location for fatty acid *β*-oxidation, where MDA was produced as a decomposition product [22]. In addition, guanine is the most easily oxidized by the hydroxyl radical among the four DNA bases, because the oxidation potential of guanine is lower than the other three DNA bases [23]. Therefore, oxidative DNA damage can produce 8-OHdG in the nucleotide pool during DNA replication [24]. Findings of high 8-OHdG levels in the organs have been considered a biomarker of DNA oxidative injury [25]. Interestingly, we found that 8-OHdG was markedly increased after weaning at d 1, d 4, and d 7, which aligned with the resulting of inhibitory hydroxyl ability (IHA) (indirectly reflected the level of the hydroxyl radical).

The classical enzymatic antioxidants, such as SOD and GSH-Px, represented a first line of defense against ROS by detoxifying them; antioxidants can remove ROS rapidly and efficiently from the intracellular environment [26]. In the present study, we found that weaning led to decreased GSH-Px but increased SOD and an upward trend at d 7 after weaning. Similarly, Yin et al. reported that plasma GSH-Px decreased, but the enzyme activity and genes of SOD in the jejunum and ileum exhibited a significant upward trend at 7 d [27]. Indeed, the increased enzymatic activity of SOD suggested the process of enhanced resistance to oxidative stress within the piglets. These results might be explained by a compensatory response to reduce the oxidative stress caused by weaning. Our results showed that weaning led to the increase of hepatic ROS and caused oxidative injury and insufficiency of antioxidant enzyme activities in the WG, which suggested a higher risk of oxidative stress than that in CG. Recently, other research suggested that oxidative stress may accompany other stresses, such as endoplasmic reticulum (ER) stress [28]. Zhao reported that ER stress arose due to the increase of X-box binding protein 1 (XBP1) expression in the hepatocyte nucleus of weaning piglets [13], which was just in accordance with our results.

To further study whether weaning resulted in hepatic apoptosis, we investigated the concentrations of caspase-3, caspase-8, and caspase-9 and the protein expression of Bax and Bcl-2. Generally, the apoptosis response is regulated by either the death receptor pathway or the mitochondrial pathway in cells, depending on different stimuli sources [29]. Apoptosis is a vital component of various processes such as normal cell turnover and chemical-induced cell death, which played an important role in oxidative stress-induced injury. Although caspases are the central components of the apoptotic response, a high Bax/Bcl-2 ratio has been found to increase induction of caspases activation, such as caspase-9, representing the mitochondria pathway [30]. However, the activation of caspase-8 was involved in the death receptor pathway. Both of these pathways activated the effector caspase-3, an executioner caspase, which initiates the process of apoptosis [31, 32]. In the present study, from the protein expression and enzyme activity, we confirmed both that the ratio of Bax/Bcl-2 and the activities of caspase-3, caspase-8 and caspase-9 were significantly increased at d 1 after weaning. Likewise, a ratio of Bax/Bcl-2 was reported, in other research, as a regulator that determined the susceptibility to apoptosis in melanoma cells [33]. San-Miguel et al. reported that the relative expression of the ratio of Bax/Bcl-2 and the activity of caspase-3 were increased in the liver of infected rabbits [34]. Recently, we have reported that weaning may induce enterocyte apoptosis through the activation of Fas-dependent and mitochondria-dependent apoptosis [7]. Taken together, the results of this study proved the hypothesis that alteration in the Bax/Bcl-2 ratio caused by weaning regulated downstream caspase-driven apoptosis.

Additionally, we detected the enzymes in liver homogenates through ELISA. ALT, AST, ALP, and GGT were abundant intracellular enzymes in the liver, which were considered to be specific indicators for hepatic damage due to subsequent leakage of enzymes into blood circulation [35]. Elevated serum ALP and GGT have been associated with damaged liver function caused by hepatic cholestasis and some destruction of the hepatic cell membranes. Both of ALP and GGT were indicators that identified bile duct obstruction or cholestasis disease, both intra- and extrahepatic [36]. In the present study, constant ALP and GGT concentrations suggested that the hepatobiliary system was unobstructed after weaning. Aminotransferases are sensitive indicators of liver cell injury and are most helpful in identifying acute hepatocellular diseases such as hepatitis. High levels of AST and ALT in the liver homogenates of the WG were found, which suggested that oxidative stress was a common mechanism that damaged hepatocellular function [35, 37]. However, further studies are warranted to explore the changes within the serum concentrations and hepatic structure of weaning piglets.

Finally, the mechanisms involved in these changes in redox status, apoptosis, and function in the liver of weaning piglets were also investigated. The MAPK pathways including ERK1/2, p38, and JNK/SAPK were considered signaling cascades for regulation of various cellular processes, such as cell proliferation and apoptosis within a wide range of cell types [14]. In our study, we found that p-JNK/JNK and p-ERK/ERK were increased after weaning, but p-p38/p38*α* showed a different fluctuation curve. This finding was not consistent with Hu et al.'s research, which reported that the ratios of p-p38/p38, p-ERK/ERK, and p-JNK/JNK were increased by weaning in jejunum [5]. However, Campbell et al. found that phosphorylated p38 was found in normal mouse livers, while it was rapidly inactivated within 30 min after a partial hepatectomy [38], which supported our results. Therefore, we believed the difference between our results and Hu et al.'s may be attributed to tissue specificity. ERKs are important for cell survival and play an adaptive role in protecting cells from oxidative stress [39]. The increased expression of ERK in this study suggested a protective effect on the livers of postweaning piglets. However, the role of stress kinases p38-MAPK and JNK in connecting redox status and apoptosis remains controversial [14]. For example, Wang et al. reported that JNK activation, but not p38, was involved in methamphetamine-induced caspase-3 activation and neuronal cell death through the mitochondrial apoptosis pathway [40]. In other research, the proapoptotic p38-MAPK activated the downstream target Bax cascade in UVB irradiated human skin [41]. These data therefore indicate that the MAPK pathways are required for proliferation and apoptosis, depending on cell types and stimuli [14]. Further studies are suggested to use the inhibitors of MAPK pathways or through overexpression or knockout of these pathways to explore whether weaning can induce hepatic apoptosis in vitro.

## 5. Conclusion

**Figure 6:**
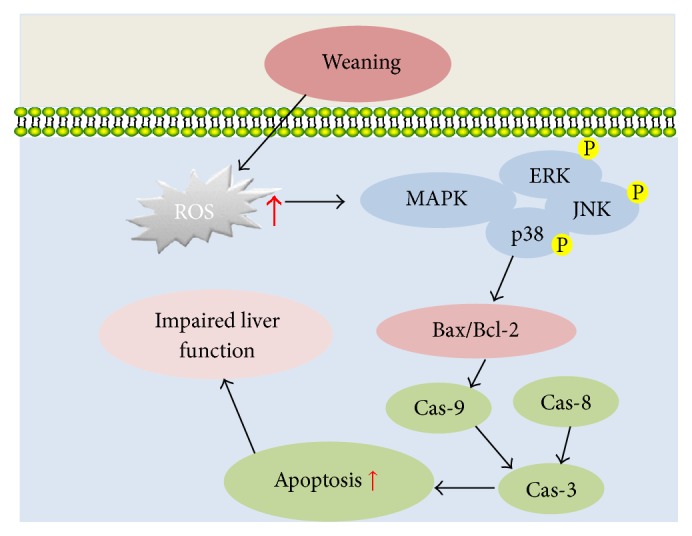
Schematic mechanisms illustrating the effects of weaning on hepatic redox status, apoptosis, and function in piglets.

Our study was the first to provide clear evidence that weaning induced hepatic oxidative stress and aminotransferases, which may be related to activated MAPK signaling pathways. A possible mechanism was suggested by this research that weaning increased the hepatic ROS production at first and then decreased antioxidant enzyme activities, which resulted in oxidative damage. Meanwhile, it initiated apoptosis by increasing the ratio of Bax/Bcl-2 and caspases involved in activating MAPK pathways (shown in Figure 6). The conclusions of this study may help to find suitable therapeutic strategies to relieve postweaning stress in both human beings and domestic animals.

## Figures and Tables

**Table 1:** Dietary ingredients and nutrient levels.

Item	Amount
Ingredients (%)	
Corn	41.18
Fermented soybean meal	5.00
Peeled soybean meal	7.00
Extruded soybean	11.22
Fish meal	5.00
Plasma protein	4.00
Whey powder	15.00
Limestone	0.50
Monocalcium phosphate	0.90
Choline	0.10
Lactose	8.75
Sodium chloride	0.35
Vitamin premix^1^	0.50
Mineral premix^2^	0.50
Total	100.00
Nutrition levels	
Digestible Energy (MJ/kg)	14.48
Crude protein (%)	20.50
Ca (%)	0.85
Total P (%)	0.67
Available P (%)	0.55
Lysine (%)	1.55
Methionine (%)	0.42
Methionine + cysteine (%)	0.83
Tryptophan (%)	0.27
Threonine (%)	1.01

^1^Provided per kg of mixed diet: vitamin A, 12 000 IU/kg; vitamin D_3_, 3200 IU/kg; vitamin K_3_, 2.5 mg; vitamin E, 80 mg; vitamin B_1_, 2.5 mg; vitamin B_2_, 6.5 mg; vitamin B_6_, 5 mg; vitamin B_12_, 0.05 mg; niacin, 45 mg; and D-pantothenic acid, 20 mg.

^2^ Provided per kg of mixed diet: folic acid, 1.5 mg; biotin, 0.15 mg; Fe, 150 mg as ferrous sulfate; Cu, 125 mg as copper sulfate; Zn, 200 mg as zinc oxide; Mn, 30 mg as manganous oxide; I, 0.3 mg as potassium iodide; and Se, 0.3 mg as selenium selenite.

**Table 2:** Concentrations of free radicals in liver homogenates of WG at d 1, d 4, and d 7 compared with CG (*n* = 5).

	CG	WG	*P*
H_2_O_2_ (*μ*mol/g prot)			
d 0	11.75 ± 0.71		
d 1	11.38 ± 0.34	14.04 ± 0.83	0.000
d 4	11.46 ± 0.69	13.49 ± 1.21	0.012
d 7	13.24 ± 1.47	14.55 ± 0.97	0.134
NO (*μ*mol/g prot)			
d 0	19.01 ± 0.64		
d 1	15.54 ± 0.32	19.46 ± 0.25	0.000
d 4	18.50 ± 1.32	19.10 ± 1.08	0.473
d 7	18.45 ± 0.90	21.11 ± 0.86	0.001

CG: control group; WG: weaning group. Differences were considered significant at *P* < 0.05.
